# Help Wanted: Science Manager

**DOI:** 10.1371/journal.pbio.0030032

**Published:** 2005-01-18

**Authors:** Kirsten A Hubbard

## Abstract

The recently created professional science master's degree may be the answer to the increasing need for science-savvy employees in the business world

“I didn't want to be just another MBA,” says Pascal Herzer, one of the first recipients of a new graduate credential known as the professional science master's, or PSM. “Not many people have the ability to understand science and business, and [the PSM] program was designed for that very purpose.”

PSMs are two-year American master's degrees financed in large part by the Alfred P. Sloan Foundation to cultivate science managers. Sloan's ultimate goal is to make science careers more attractive to talented young people like Herzer, a 2003 PSM graduate in Applied Biosciences from the University of Arizona, who believes his PSM makes him more marketable to science-based businesses. “I am at the true junction of science and business,” he says.

## The Missing Degree

Fortunately for Herzer, the business of science is booming. Jobs for scientists and engineers grew four times faster than the United States national average since 1980, and should outpace the market until at least 2010. Surprisingly to many academics, most of these jobs are in industry. In 1999, the last year with complete data, two out of three employed science and engineering (S&E) graduates worked in industry, including the great majority of bachelor's and master's degree holders, and 40% of doctorates. In other words, industry, not academe, now drives American S&E employment, and will for the near future.

Like academia, industry needs scientifically literate personnel; unlike academia, industry wants employees with business savvy as well. However, in the past, graduate students received either science or business instruction, not both. “Industry simply hired regular master's-degreed people, or MBAs, or more likely PhDs, and just expected them to learn their weaknesses on the job,” says Eleanor L. Babco, Executive Director of the Commission on Professionals in Science and Technology, a nonprofit corporation with funding from the Sloan Foundation to assess PSM graduates.

For science-based businesses, then, the American S&E doctorate—viewed by many as the worldwide gold standard for science education—is too specialized for their needs (see Box 1). But a master's degree may be just right.

**Box 1. Is There a Doctorate in the House?**

The length of time to obtain a biological science doctorate has increased…[Fig pbio-0030032-g001]

…The number of postdocs and part-time faculty in the biological sciences is increasing…[Fig pbio-0030032-g002]

…And the proportion of doctorates in academia is decreasing…[Fig pbio-0030032-g003]

(Statistics taken from the National Science Board's “Science and Engineering Indicators 2004” [http://www.nsf.gov/sbe/srs/seind04/] and the National Science Foundation Science Resources Statistics Division's “1995 and 2001 Survey of Doctorate Recipients” [http://www.nsf.gov/sbe/srs/infbrief/nsf04328/table1.xls].)

## Bridging the Gap

During the 20th century, the master's degree evolved as a professional credential in many fields, including business, education, and social work, and more recently, pharmacy, physical therapy, and accounting. In the 1990s, non-incidental master's in the sciences—in other words, intentionally terminal degrees, not consolation prizes for failing out of graduate school—crept into engineering and applied mathematics, too, as companies grew more reliant on computational analysis and hired accordingly. From 1981 to 2000, for example, the number of earned master's degrees in mathematics and computer science more than doubled.

With hopes of spurring a “significant movement,” in 1997 the Sloan Foundation bet big on professional master's degrees, eventually spending $11 million on almost 100 programs across the US. Sloan Foundation–backed PSM programs now operate at 45 universities in 20 states, in such fields as microbial biotechnology and applied genomics; similar programs have also developed independently of the Sloan Foundation, such as the Master of Science in Bioinformatics at Johns Hopkins. And while most PSM-style programs are currently in the US, this may soon change: the 1999 “Bologna Agreement” requires all European Union universities to adopt uniform undergraduate and graduate degrees “relevant to the European labour market”; so master's-level industry-centric degrees are sure to follow. At Leiden University in the Netherlands, for example, students can now add a “science-based business” focus to any research master of science (MSc) program.

Like all graduate programs, PSMs offer advanced coursework in a (science or math) specialization, usually in an emerging or hybrid field such as bioinformatics. Most PSMs also provide business courses—including finance, project management, regulatory affairs, and intellectual property law—and information technology classes as well. PSMs are “industry relevant” by design, with external advisory committees populated by local business leaders, weekly colloquia led by corporate representatives, special arrangements for employed students, and industry internships or final projects exploring realistic business scenarios (see Box 2).

**Box 2. Requirements for PSM Programs**

Only programs meeting most of the following requirements may earn the official moniker “professional science master's.”

- Two years of science or math graduate-level coursework, taught by regular faculty, characterized by interdisciplinary studies and a focus on informatics
- Training in business fundamentals—such as finance, marketing, project management, communication, and team building—and exposure to industry professionals
- Final project reflecting a realistic workplace issue and/or industry internship
- Advisory board of industry professionals
- Targeted recruitment and admissions separate from other degree programs
- Commitment to tracking graduates through first five years
- Long-term sustainability

(Source: http://www.sciencemasters.com/affiliation.html.)

A key principle underlying the PSM model is interdisciplinarity. PSM students are encouraged to reach out to other departments and broaden their expertise in multiple areas, to better understand the collaborative culture of industry-style scientific enterprise. To promote such connections, PSM programs explicitly teach teamwork and effective scientific communication, with authentic case studies analyzed alongside MBA students, classroom presentations and public seminars, and open defenses of final projects. Consequently, PSM graduates, unlike many doctoral graduates, are trained to possess a wide array of interactive skills, including sizing up an audience for their ability to comprehend the presented material and adapting appropriately.

In a science-based business, ideas must flow freely between scientists and non-scientists in and out of the company—between researchers and marketers, say, or inventors and patent lawyers—to capitalize on discoveries and comply with regulations. When non-scientists misunderstand the science underpinning a business model, profits suffer. But the presence of a central employee who streams data between differently educated members of the network may boost the bottom line. PSM students are specifically trained to act as such “science translators.” “[My PSM] allows me to serve as an efficient mediator between corporate entities, university personnel, and scientists,” says Herzer.

For this reason, small companies and start-ups, which cannot afford specialists for every position, may particularly benefit from PSM-credentialed employees, able to connect different people and function in multiple roles; indeed, many PSM graduates have job descriptions expressly created for them. “We need generalists rather than specialists,” says James L. Ratcliff, Chairman and CEO of Rowpar Pharmaceuticals, a dental products company in Scottsdale, Arizona. For small companies like his, Ratcliff says, “PSM graduates have an appropriate combination of project management expertise, an understanding of business environments and priorities, and advanced knowledge in the physical and life sciences.”

Although it is too early for comprehensive assessment, employment outcomes for PSM graduates have been examined, and this result is clear: they are getting industry jobs. According to The Conference Board, an independent business management organization funded by the Sloan Foundation to survey PSM alumni, by 2002, 91% of the first PSM graduates had obtained full-time positions within their field despite a white-collar recession, two-thirds with salaries of $50,000 or more. A separate analysis by the Commission on Professionals in Science and Technology found that 61.5% of employed respondents were hired by businesses. Employment opportunities range from marketing to bioinformatics (see Box 3). “Companies need people that can work in companies,” says Lindy A. Brigham, coordinator of the Applied Biosciences PSM program at the University of Arizona.

**Box 3. First Jobs Obtained by PSM Graduates**

A sampling of actual first jobs obtained by students in PSM or PSM-style programs:

- Coordinator of Regulatory Affairs
- Associate Criminalist
- Licensing Assistant
- Staff Researcher
- Senior Computer Database Specialist
- Project Manager
- E-Product Marketing Specialist
- Clinical Consultant
- Technical Support Specialist
- Bioinformatics Programmer
- Manager of Medical Affairs

(Source: personal communications from L. A. Brigham, D. Ascher, T. Tiongson Pohar, and S. Inamdar.)

## Not a Perfect Cure

Although most scientific careers demand a graduate degree, a professional master's in many hard sciences still encounters entrenched academic opposition. According to Lee-Jen Wei, then acting chair of the Department of Biostatistics in the Harvard School of Public Health, quoted in the *Wall Street Journal*, “Harvard tries to create leadership in industry, academics and government, and our philosophy is we don't think that with a master's degree people can fill that role very easily.”

The government appears to agree with this view. While most doctoral candidates receive federal funds for tuition and other expenses, there is little money for master's students, who disproportionately end up in industry regardless of specialization. PSM students are especially affected by this problem because “interdisciplinary” equals “expensive.” Similarly, “interdisciplinary” can also mean “hard to find”—companies with targeted recruitment often miss PSM students, who are not “in” any particular department—and “confusing”—differences in these new, still somewhat vaguely defined programs can make hiring comparisons difficult. But perhaps the most conspicuous drawback to PSMs is their newness, and resulting obscurity: almost half of graduates say they are “not sure” employers will value their PSM, or the unique skill set it affords.

## But Will They Succeed?

Still, many observers of higher education support the PSM concept. Judith Glazer-Raymo, author of the forthcoming book *Professionalizing Graduate Education: The Master's Degree in the Marketplace*, argues that converging market forces will lead to the success of the professional master's degree in science. These forces include: rapid technological change; the rise of alternative learning channels such as online and distance education, corporate universities, and hi-tech certification programs; the proliferation of degrees in general, and in multidisciplinary fields specifically; and a fundamental societal shift away from public service and toward entrepreneurship, profitability, and competition.

Kenneth R. Smith, former dean of the Eller College of Business and Public Administration at the University of Arizona, and others make the case that PSMs may protect students' careers from outsourcing to foreign countries. The American S&E labor pool is shrinking, and industry has already responded by transferring much of its research and development overseas; however, companies are mostly moving lab scientists, not strategic analysts. Cross-training in both science and business could thus provide an edge for domestic workers in the near-term employment environment; in fact, PSM programs have a higher proportion of US citizens and residents than S&E doctoral programs.

Further, in its 2003 report, the National Science Board urged the government to better align S&E graduate education with “expected national skill needs,” including “interdisciplinary skills.” The report also recommended federal funding for a wider range of educational options and more attention on the real economic concerns of students—code words for support of professional master's degree initiatives. In the same vein, top universities now advocate “interconnections” between their professional schools and traditional departments, as a way of strengthening the overall academic mission, and many countries are sponsoring initiatives to stimulate university–industry links, to maximize marketing of technological innovations.

For advocates, then, the PSM both advances the cause of science education reform and addresses changing employment conditions with one big idea: reinvention of the two-year graduate credential for an entrepreneurial age. Herzer, for one, now a technology development representative at the Scripps Research Institute, has staked his future on the potential of professional master's degrees. “Scientists rarely understand business dealings, and business personnel rarely comprehend scientific discoveries,” he says. “The overlay of the two is crucial for any successful business transaction of scientific origins.”

**Figure pbio-0030032-g001:**
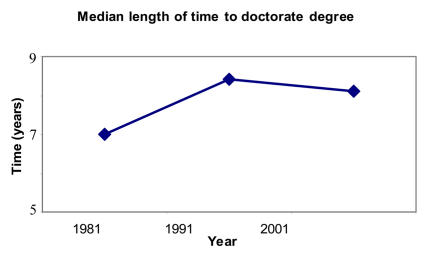
Median length of time to doctorate degree

**Figure pbio-0030032-g002:**
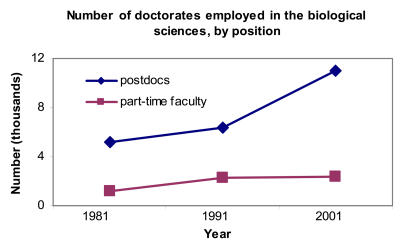
Number of doctorates employed in the biological sciences, by position

**Figure pbio-0030032-g003:**
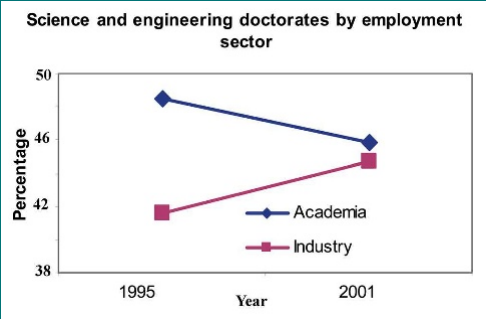
Science and engineering doctorates by employment sector

## Abbreviations

PSM: professional science master's
S&E: science and engineering

